# Steering the Private Sector in COVID-19 Diagnostic Test Kit Development in South Korea

**DOI:** 10.3389/fpubh.2020.563525

**Published:** 2020-11-17

**Authors:** Sora Lee

**Affiliations:** Menzies Centre for Health Governance, School of Regulation and Global Governance (RegNet), ANU College of Asia & the Pacific, The Australian National University, Canberra, ACT, Australia

**Keywords:** COVID-19, governance, infectious disease, test kit, private sector, biotech industry, South Korea

## Abstract

Responsive private sector engagement in developing test kits for coronavirus disease (COVID-19) in South Korea offers a valuable case study in public–private partnership and infectious disease governance. Korean biotech firms promptly developed diagnostic test kits, and the nation achieved capacity to test more than 20,000 people daily. This was a direct result of the continuous application of lessons learned from the Middle Eastern respiratory syndrome outbreak in 2015. South Korea had been strengthening the private sectors' infectious disease governance and response capacity, creating various new constructive pathways toward public–private partnership. Regulatory amendments were made to better liaise with the private sector. Government-led investment had increased in the research and development of testing technologies over the past 5 years. Furthermore, the Korean government had introduced fast-tracking approval, allowing open competition for more than 20 domestic biotech companies to develop test kits. An overview of test kit governance informs us of the importance of public–private partnership for pandemic threats.

## Introduction

South Korea was one of the most severely hit nations in the early days of the coronavirus disease (COVID-19) outbreak. South Korea experienced a rapid increase of positive cases in the first 2 weeks of its outbreak, reaching more than 800 new cases in late February (1). Since then, the number of new cases dropped steadily, and the country successfully suppressed the disease without restricting movement of people or having long lockdowns. While it is still too early a stage of COVID-19, evidence indicates that the curve of cumulative confirmed patients in Asia is becoming flatter (2). As the virus continues to spread, communicating country-specific responses is critical for countries that have not yet prepared for such severe risks or those who are currently struggling to control the virus. Numerous articles have shed light on South Korea's effective measures to contain the virus when hit by a rapid, exponential increase in infections. Underlying these effective actions was a consistent and coherent strategy to “nurture private capacity and partnership,” paving ways that enabled rapid COVID-19 testing. As Huang from the US Council on Foreign Relations says, “South Korea's experiences showed how sound coordination between the state and private sector can benefit efforts to screen and contain the disease.”^1^ It was not a question of state-of-the-art scientific knowledge, rather, a governance question to allocate, and liaise with, existing and potential resources, especially from the private sector.^2^

## Middle Eastern Respiratory Syndrome–Invoked Changes in South Korean Infectious Disease Governance

After the COVID-19 outbreak, government assigned clear responsibilities to the private sector for prevention and containment, on-the-ground responses, treatment, and quarantine in South Korea. This was achieved through rigorous implementation of established public health resources, widely available and accessible testing,^3^ rigorous contact tracing using big data,^4^ and innovation in technologies (11). Since the early phase of the spread, South Korea formed a tight network of screening. More than 18 laboratories and 633 testing sites, including drive-through clinics, ensured fast and affordable public testing. This mass testing was one of the drivers that resulted in early and effective quarantining. This would not have been possible if the nation suffered from a shortage of test kits. Korean biotech firms promptly developed diagnostic test kits, and the nation obtained the capacity to test more than 20,000 people daily (12).

The rapid development of test kits was possible because of the Middle Eastern respiratory syndrome (MERS) outbreak in 2015. The nation learned a painful lesson of quarantining following the 2015 outbreak of MERS. A single imported case of MERS prompted a chain of transmissions in a private hospital, with 186 infected cases and 36 deaths, the highest number anywhere outside the Middle East region. This resulted in the quarantining of 17,000 people, with the government harshly criticized for its slow response. The massive changes in regulations in infectious disease governance occurred after MERS in South Korea (8). An intensive investment in the biotech industry and systematic building of public–private partnership occurred as a result of MERS. The Table 1 shows the comparison of key indicators of infectious disease governance between the MERS and the COVID-19.

**Table 1:** Comparative table on infectious disease governance between MERS and COVID-19.

	**MERS**	**COVID-19**
Status of KCDC	Limited authority	Expanded authority and responsibility
Emergency approval	Not available	Available
Private sector testing	No fast-track approval system for commercial diagnostic kits	Active
Department in charge of testing	No specific division in charge	Division of Laboratory Diagnosis Management, Center for Disease Control and Prevention

The Infectious Disease Control and Prevention Act, initially enacted in 1954 but revised in 2016, provides government with the necessary powers to distribute resources and engage with a wide range of actors to effectively stop disease transmission. According to Lee (13), the Act was to prepare for future unexpected infectious disease threats. The Act was set up with the purpose of improving communication and coordination in the event of infectious disease outbreaks. The Act specifies responsibilities and accountabilities of the KCDC to exert a certain level of control over regional governments, the private sector, medical practitioners, and the public. The expansion of power and regulatory authority for KCDC has allowed a rapid response from KCDC as a control tower.

## Private Sector Engagement for COVID-19 Testing

**Figure 1:**
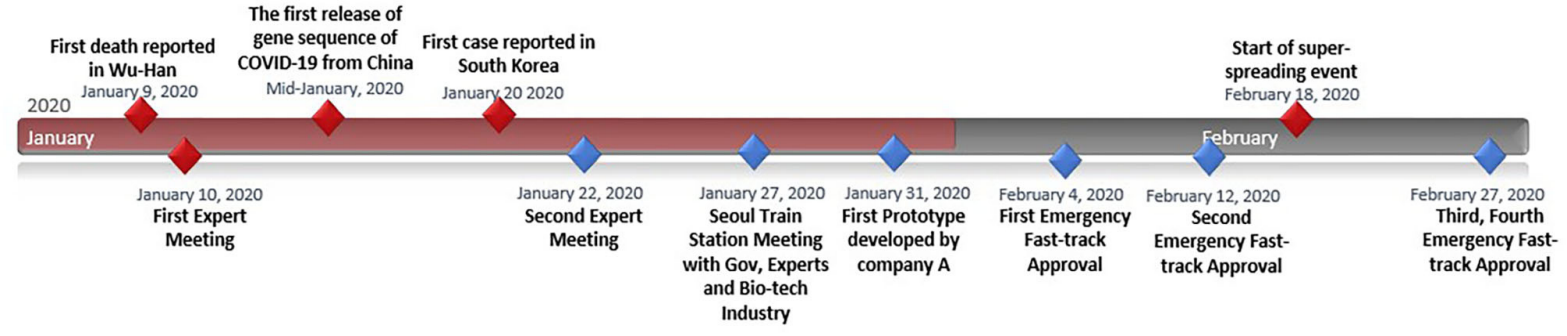
Timeline of COVID-19 test kit development (14–17).

As seen from the previous section, the conditions did not arise in a vacuum. Actions and changes occurred from different levels and directions after the MERS crisis. Perhaps the single most important actor that South Korea invested in rigorously and continuously was the private sector. The government's direction was strategic with the clear goal to nurture the capacity of the private sector. Such actions enabled an effective public–private partnership for COVID-19 governance. The key events and the timeline are illustrated in Figure 1 to show the efficacy of governance responses.

### Research and Development and Private Sector Capacity

Policies addressing the private sector's role in infectious disease control have gone through significant changes since the MERS outbreak. The biggest lesson for South Korea was that the state of medical care and quarantine were two separate issues. The medical facility (a private hospital), with state-of-the-art medical knowledge and technologies, failed to quarantine, thereby becoming the source of transmission of the extremely contagious MERS. According to Lee (13), the necessity for strong political will and budget expansion on quarantine became the prevailing thoughts of public managers. In 2016, the budget for contagious diseases and quarantine systems was expanded by 134% compared to the previous year, a jump from US $58 million to $135 million. In 2020, the budget has continuously risen with an increase of 182% over the last 5 years.

The biggest investment occurred in “preventive measures for newly contagious diseases,” that is, the purchasing of antiviral products and personal protective equipment (US $37 million). The second highest expenditure was for the “development of preventive treatment technologies for newly contagious diseases” and comprised US $21 million. This was used to fund research and development (R&D) projects for vaccines, preventive technologies, and test kits. The top five budget allocations were for preventive measures and R&D projects, such as “technology development for contagious diseases management,” “crisis response technology development,” and “establishment and management of a public vaccine development support center.” The sum of R&D projects amounted to US $67 million of US $135 million, with 49% of the entire budget on infectious diseases. In 2016, the quarantine management budget rose to US $6.6 million, up from US $3.4 million in the previous year (8). This reflected the government's position that quarantine failure was the direct reason for the 2015 MERS outbreak.

South Korea has a burgeoning biotech industry that comprised scientist-led small-sized entrepreneurial startups. Korea has nurtured R&D-based bio ventures through strong political will and a vision of global markets. After MERS, experts realized that the best strategy to fight infectious diseases was to test early as the development of treatments or vaccines takes time. Therefore, the government actively encouraged companies to acquire the necessary technology to enable quick diagnosis and easy applicability across multiple sites. While criticism of budget expenditure exists, the Korean government recently declared an extra US $8 million investment in small and medium entrepreneurial companies to support the development and production of test kits (15). This will further allow the active participation of the biotech industry in seizing business opportunities.

Although biotech companies have developed tests and manufactured equipment, it is the laboratories in universities, hospitals, and government agencies that have played a crucial role during the COVID-19 crisis in South Korea. The Korean Society for Laboratory Medicine (KSLM) is the key actor enabling laboratory preparedness and responsiveness to the infectious disease pandemic. The groundwork for the partnership between laboratories and the KCDC was set during the MERS outbreak. A national accreditation system has since been established by the KCDC for infectious disease laboratories to ensure a consistent response (18). Numerous scholars have predicted the importance of the KSLM maintaining and enhancing laboratory responses in future crises and essential to deploying consistent and coherent nationwide guidelines for laboratory diagnostic tests (19, 20). As will be discussed in the following section, the KSLM also contributed to maintaining the quality of diagnostic testing for prototype test kits developed by biotech firms. KSLM provided unbiased validation sites and procedures crucial to promoting the rigor of KCDC's fast-track approval process (21).

### Emergency Approval Process

The KCDC used emergency procedures to fast-track the development of test kits. In the very early phase of the COVID-19 spread, South Korean health officials screened the nation's biotech firms, based on their expertise and outputs, and invited 20 or so companies to a task force meeting.^5^ The government's urgent call for test kit development was delivered to industry partners so that the country was equipped with an effective testing capacity. KCDC shared its knowledge about the virus with these companies and announced emergency fast-track approval for those making test kits.

The Ministry of Health and Welfare communicated to the public, the process by which the private sector would develop testing kits through a policy briefing (15). The Korean government started an early series of expert meetings. One week after the first meeting, KCDC had their first diagnostic test kit prototype. Other firms joined in, one after another. KCDC was well aware that initial test kits may be of low quality, given the short period for development. Thus, the KCDC embarked on mass cross-checking of the initial pool of patient samples. Cross-checking involved more than 100 laboratories nationwide confirming the accuracy of the test kits. The KCDC disclosed all information on test methods conducted in order to assist test kit companies. The government's message was clear, decisive, and supportive of the companies. After rapid but rigorous testing, the government announced its first approval on February 4, 2020. A second company received government approval on February 12, 2020, for their product. Shortly after, the nation was hit by a large spread of the disease in the city of Daegu. On February 27, the country acquired two more approved test makers according to the KCDC. More than 654,863 people had been tested as of May 8, 2020 (22). This allowed the biotech industry to share abundant samples to improve test kit accuracy. Korea conducts up to 15,000–20,000 tests a day, with the remainder exported to other countries.

According to *The Diplomat* (23),^6^ South Korea's major producers of COVID-19 tests were expected to export up to 5 million test kits per week in May. The sum total of South Korean COVID-19 test kit export rose sharply from US $50–$132 million, to more than 60 countries in the first 20 days of April.^7^ Aside from commercial revenue, the South Korean government collaborated with the private sector to donate kits for diplomatic and aid purposes. According to the Ministry of Foreign Affairs, 117 countries have asked for kits as humanitarian aid or to import. Of these 117 countries, 37 countries are currently liaising with Korean partners and networks. Officials confirm that additional aid would be provided to remainder countries, based on bilateral relations and partner country's capacity on public health infrastructure (25).

## Conclusion

This article focuses on the public–private partnership strategy as one of South Korea's critical enablers of COVID-19 test kit development. The effective steering of the private sector required regulatory preparation, investment, and political decisiveness. The process of weaving the capacities of both public and private actors had been continuous and coherent since 2015, as seen from this analysis. Significant changes in regulation maximized private capacity for disease control. Massive grants available to the biotech industry for testing technologies provided fertile soil. Following the COVID-19 outbreak, companies were given all the information and support in open competition under emergency fast-tracked approval processes. Simultaneous massive public testing reinforced the technologies of biotech companies through reliable data, improving their inventions. COVID-19 exports of testing kits and personal protection suits increased sharply, uplifting the entire industry, coupled with the development of treatments, vaccines, and other related areas.

It may be too soon to evaluate South Korea's steering of public–private partnership as a more or less effective response to COVID-19, as the battle against COVID-19 continues. Nonetheless, the South Korean COVID-19 response in the public–private governance context can guide the long-term governance strategy of other countries by enabling collective and coherent responses from the private sector as they prepare for the continued threat. The essence of the South Korean case is the process of coordination that reduced the gap between private and public sectors and public interest in the collaboration, which can be intuitively applied to various countries. Furthermore, this article will be particularly relevant for countries with relatively higher portions of private medical care and active public investment in the fast-growing biotech industry, such as Turkey, Brazil, and India. It may be timely for scholars worldwide to engage in discussion on the evolvement of public–private readiness in global COVID-19 governance. The virus reminds us how interdependent we are as individuals and as a nation. International knowledge transmission and reciprocal learning processes on COVID-19 are vital.

## Data Availability Statement

All datasets presented in this study are included in the article/supplementary material.

## Author Contributions

The author confirms being the sole contributor of this work and has approved it for publication.

## Conflict of Interest

The author declares that the research was conducted in the absence of any commercial or financial relationships that could be construed as a potential conflict of interest.

## Footnotes

^1^As shown by another spike of new cases in early May, driven by Seoul's Itaewon district night clubs, the pandemic is far from over. This article focuses on test kit development, which allowed South Korea's strategy to test, trace, and quarantine. For more information, see (3).

^2^A brief description of public accountability of private medical institutions in South Korea can be detailed as follows. Under the Public Health and Medical Services Act, last amended in 2012, it is the responsibility of State, local governments, and public and medical institutions that provide “public health and medical services, to effectively provide citizens with higher-quality public health and medical services and contribute to the improvement of national health by prescribing basic matters regarding public health and medical services. (4).” The public interest in hospitals was often considered as public control that impedes private value–profit maximization (5). Others view that the Act can very much be used to outsource public health to the private sector rather than coordinate and regulate (6). Nonetheless, there has been a sizable budget coming from the public sector that needs to be accounted for. So far, the publicness of private sector has only been accounted narrowly for the three main areas: quality of medical services provided, health inequity, and unmet health needs (7).

^3^The testing procedures are detailed in (8).

^4^Smart Management System (SMS) is a regulatory solution that government chose as an innovative technology for epidemiological tracing, which significantly reduces administrative inefficiencies by collaborating data from multiple agencies. See (9). Nonetheless, there is a criticism that it would be more appropriate to train more local field epidemiology officers (10).

^5^The implementation of public–private partnerships has not always been positive. A small number of biotech firms who complained about the government's crude criteria for firm selection were excluded during test kit development. The government then reaffirmed funding and support for companies who had not been preselected, but expressed their interest in test kit development.

^6^For information regarding usage of testing kits as humanitarian aid, please visit https://thediplomat.com/2020/04/how-south-korea-is-building-influence-through-covid-19-testing-kits/.

^7^The countries may have overlapping actors involved, from private sector engaged in the community, to official assistance using formal diplomatic channels. For more information, see (24).
